# Cannabis in medicine: a national educational needs assessment among Canadian physicians

**DOI:** 10.1186/s12909-015-0335-0

**Published:** 2015-03-19

**Authors:** Daniel Ziemianski, Rielle Capler, Rory Tekanoff, Anaïs Lacasse, Francesca Luconi, Mark A Ware

**Affiliations:** 1 Alan Edwards Pain Management Unit, McGill University Health Center Research Institute; Canadian Consortium for the Investigation of Cannabinoids, A5.140 Montreal General Hospital, 1650 Cedar Avenue, Montreal, Quebec H3G 1A4 Canada; 2 Interdisciplinary Studies Graduate Program, University of British Columbia, Vancouver, Canada; 3 Département des sciences de la santé, Université du Québec en Abitibi-Témiscamingue, Québec, Canada; 4 Continuing Health Professional Education, Faculty of Medicine, McGill University, Montreal, Québec, Canada; 5 Community Programs, Urban Care Health Group, Toronto, Ontario Canada

**Keywords:** Cannabis, Medical marijuana, Needs assessment, Continuing medical education, Health professionals

## Abstract

**Background:**

There is increasing global awareness and interest in the use of cannabis for therapeutic purposes (CTP). It is clear that health care professionals need to be involved in these decisions, but often lack the education needed to engage in informed discussions with patients. This study was conducted to determine the educational needs of Canadian physicians regarding CTP.

**Methods:**

A national needs assessment survey was developed based on previous survey tools. The survey was approved by the Research Ethics Board of the McGill University Health Centre Research Institute and was provided online using LimeSurvey®. Several national physician organizations and medical education organizations informed their members of the survey. The target audience was Canadian physicians. We sought to identify and rank using 5-point Likert scales the most common factors involved in decision making about using CTP in the following categories: knowledge, experience, attitudes, and barriers. Preferred educational approaches and physician demographics were collected. Gap analysis was conducted to determine the magnitude and importance of differences between perceived and desired knowledge on all decision factors.

**Results:**

Four hundred and twenty six responses were received, and physician responses were distributed across Canada consistent with national physician distribution. The most desired knowledge concerned “potential risks of using CTP” and “safety, warning signs and precautions for patients using CTP”. The largest gap between perceived current and desired knowledge levels was “dosing” and “the development of treatment plans”.

**Conclusions:**

We have identified several key educational needs among Canadian physicians regarding CTP. These data can be used to develop resources and educational programs to support clinicians in this area, as well as to guide further research to inform these gaps.

## Background

Canadian patients have had access to cannabis for therapeutic purposes (CTP) under Health Canada’s Medical Marihuana Access Program (MMAP) since 1999. As of June 2013, over 30 000 Canadians had licenses to possess CTP; this was projected to reach approximately 50 000 in 2014 and 400 000 in 2024 [1]. The new Marihuana for Medical Purposes Regulations (MMPR), which came into effect on 19 June 2013, completely replaced the Medical Marihuana Access Regulations (MMAR) on April 1 2014 [2]. Under the MMPR, patients may be authorised to possess herbal cannabis if they are issued a valid medical document from either a physician or a nurse practitioner; the medical document is not strictly speaking a ‘prescription’ as cannabis is not an approved drug, but it does contain information on daily ‘dose’ of cannabis (in grams/day) and duration of validity. No diagnosis is required as there is no formal ‘indication’ for CTP. The MMPR therefore maintains the physician’s pivotal role in patients’ access to CTP, despite concerns expressed by physicians about insufficient information on the risks and benefits of CTP, insufficient information regarding the appropriate use of CTP [3,4] and insufficient information with which to compare CTP with pharmaceutical cannabinoids.

Prior physician surveys have explored opinions about cannabis legalization [5-8] and attitudes towards CTP [9,10]. CTP-related surveys have been directed at specific physician populations, including oncologists [6-8] or family physicians [9]. The need for further medical education and training on CTP has been reported by Colorado family physicians [9] and American oncologists [6]. An understanding of the current and desired status of Canadian physicians’ educational needs on CTP remains to be quantified. We therefore conducted an educational needs assessment among Canadian physicians to quantify perceived knowledge levels and identify knowledge and practice gaps. This survey was also designed to explore Canadian physicians’ experiences and attitudes towards CTP, to list perceived barriers to the use of cannabis as a possible treatment option in clinical practice, and to make recommendations for the preferred format of physician education on CTP. The study was conducted in order to inform strategies to overcome knowledge and practice gaps, to increase competence and to improve patient care in this complex and controversial area.

## Methods

We conducted an online survey of Canadian physicians from November 2012 to March 2013. Physicians were contacted through existing medical and health care organizations, which were asked to distribute the survey to their members. Direct email invitations to physician members were sent by five organizations, and links from organization websites to the survey were provided by three organizations. Electronic invitations included a summary of the survey, consent information and a link to access the online survey.

Survey questions were adapted from prior needs assessment surveys distributed by the Canadian Consortium for the Investigation of Cannabinoids (CCIC) between 2009 and 2012 [4]. The research team reviewed the survey for construct validity and four physicians (a rheumatologist, anesthesiologist, internist, and pain specialist) pilot-tested the survey. Limesurvey® (https://www.limesurvey.org/en/) was selected to host the survey as it is supported by McGill University and data are securely stored within the institution. The study protocol was approved by the McGill University Health Centre Research Institute Research Ethics Board.

The survey consisted of six sections. The first section concerned knowledge factors – respondents were asked to rank their perceived current and desired level of knowledge on 9 CTP-related topics (see Table 1) using a 5-point Likert scale (1: very poor; 5: very good). They were also asked to rank how strongly they felt the need for education on CTP using a 5-point Likert scale (1: not at all; 5 very strongly). The second section addressed experience – five questions with binary responses (yes or no) explored physicians’ clinical experiences with pharmaceutical cannabinoids, the federal MMAR process and discussions regarding CTP with their patients. The third section addressed barriers – a list of potential barriers regarding the use of CTP were offered, from which the respondents could select one or more, as well as the opportunity to indicate any other obstacles to the use of CTP in their practice. The fourth section concerned attitudes – respondents were asked to specify which health care professionals, if any, they felt should be authorized to approve CTP for patient use. They were also asked to rank, using a 5-point Likert scale (1: strongly agree; 5: strongly disagree), how strongly they agreed with a series of statements about personal comfort levels with prescribing or authorizing CTP. The fifth section addressed educational approaches - eleven commonly used educational methods were listed from which respondents were asked to select their preferences, along with an opportunity to provide other alternatives or comments. Finally, the sixth section requested demographic information - respondents were asked to indicate region, setting and focus of their practice, and number of years in practice. The survey took an estimated 10–15 minutes to complete.

**Table 1:** **Analysis of knowledge scores and gaps for therapeutic use of cannabis (ranked by gap size)**

Knowledge area	Mean current knowledge score (1–5)	Mean desired knowledge score (1–5)	Mean GAP ^ 1 ^
Dosing and creating effective treatment plans for patients using medical cannabis	2.25	3.95	1.78
Similarities and differences between dried cannabis, other forms of cannabis products, and prescription cannabinoid medications	2.36	4.00	1.70
Health Canada’s Marihuana Medical Access Regulations (MMAR) Program	2.43	3.99	1.60
Laws and regulations surrounding the medical use of cannabis	2.65	4.11	1.49
Safety, warning signs and precautions for patients using medical cannabis	2.84	4.21	1.48
Alternative routes of administration of medical cannabis	2.72	4.02	1.42
Mechanism of action of cannabis (endocannabinoid system)	2.78	4.06	1.39
Potential risks of using cannabis for medical purposes	3.06	4.23	1.28
Potential therapeutic uses for cannabis	3.07	4.17	1.22

^1^Gap is calculated (using individual response pairs) = (desired knowledge level -current knowledge level).

### Data analysis

Descriptive statistics (frequencies and percentages) were used to summarize respondents’ knowledge, experiences, barriers, attitudes, preferred educational approaches, and demographic information. Open-ended comments were reviewed for common themes, sorted and counted. Data were entered and analysed using Microsoft Excel® (Redmond, USA).

The difference between current and desired knowledge levels was used to determine a perceived knowledge gap. The knowledge gap was calculated based on how much greater the individual, not average, desired knowledge level was compared to their current knowledge level. Only response pairs were used for the calculation; responses only to the current or desired question were excluded, and responses where the indicated desired level was lower than the current level were also excluded.

## Results

### Participant demographics

Eight of 15 organizations contacted agreed to distribute the survey to their members or to post the survey on their websites. Participating organizations were the Canadian Association for HIV Research, the Canadian HIV Trials Network, the University of British Columbia Faculty of Medicine Office of Continuing Medical Education, the Canadian Association for Physical Medicine and Rehabilitation, the Canadian Consortium for the Investigation of Cannabinoids, the McGill University Continuing Professional Development Office, MedCanAccess, and RxMedia Healthcare Communications Inc. Based on the estimated size of organisational mailing lists and organisation member outreach, we estimated that a total of 25,298 invitations to participate were sent. The survey was accessed a total of 580 times. From the total number of times accessed, 108 did not proceed past the introduction and consent page, 19 viewed the survey but did not provide any responses, and 27 did not submit their responses. A total of 426 usable and complete responses were received.

Demographic characteristics of respondents are presented in Table 2. Just over half of the respondents (54%) were physicians with 21 years or more in practice. While we do not have national data on the distribution of physicians by years of practice, 42% of physicians are over the age of 55y in Canada, and 68.1% are over 45y, suggesting that our sample is roughly consistent with national physician demographics [11]. The survey was completed in English by 91% and in French by 9% of respondents. Respondents’ region and setting of practice were roughly proportional to the Canadian national physician distribution [12].

**Table 2:** **Demographic characteristics of needs assessment respondents (n = 426)**

Characteristic	n	%
**Area of practice**		
GP/FP	117	27
GP/FP with enhanced area	72	17
Specialist	219	51
Other^1^:	6	1
Not specified	12	3
**Number of years in practice**		
0 to 5	40	9
6 to 10	39	9
11 to 15	45	11
16 to 20	59	14
21 or more	230	54
Not specified	13	3
**Region of practice**		
Atlantic	38	9
Quebec	100	23
Ontario	128	30
Prairies	70	16
BC	64	15
Territories	6	1
Not specified	20	5
**Community where practicing**		
Urban	226	53
Rural	92	22
Both	96	23
Not specified	12	3
**Language of survey completion**		
English	388	91
French	38	9

^1^Resident (2), Medical Advisor (2), Not Specified (2).

### Knowledge

The perceived current and desired knowledge levels on 9 CTP related sub-topics are shown in Table 1. The lowest average current knowledge levels were found for dosing and creating effective treatment plans for patients using CTP (2.25/5) and similarities and differences between dried cannabis, other forms of cannabis products, and prescription cannabinoid medications (2.36/5). The highest average desired knowledge levels concerned potential risks of using CTP (4.23/5) and safety, warning signs and precautions for patients using CTP (4.21/5); for these topics, 87.5% and 87.3% of respondents desired a good or very good level of knowledge respectively. The largest gap between perceived current and desired knowledge levels was identified for dosing and the development of treatment plans (average gap = 1.78) followed by comparison of cannabis, cannabis products and prescription cannabinoids (average gap = 1.70) and knowledge of the regulatory framework (average gap = 1.60). The need for education on cannabis in medicine was reported as strong or very strong by 64% of respondents, compared to 19% who were neutral, and 17% not very strongly or not at all.

### Experiences

Most respondents (79%) reported having been approached by a patient and/or his/her family to discuss the use of CTP, while 39% reported initiating a discussion with a patient and/or his/her family on the use of CTP. Two-thirds of respondents (66%) reported having patients using CTP, while 36% reported having ever signed a medical declaration for the MMAR. Experiences with pharmaceutical cannabinoid medications were varied. Of the 59% who had ever prescribed a cannabinoid, nabilone was the most common (51%), followed by dronabinol (19%) and nabiximols (18%), while 41% of respondents reported having never prescribed a pharmaceutical cannabinoid.

### Barriers

The list of barriers to the use of CTP are shown in Table 3. The most common was a concern that patients who request CTP may actually want it for recreational purposes (65%), while a lack of guidelines for the clinical use of cannabis and the need for more data on risks and benefits were reported by 64% and 56% of respondents, respectively.

**Table 3:** **Perceived barriers regarding the use of CTP**

Factor	n ^ 1 ^	%
Concern that patients who request medical cannabis may actually want it for recreational purposes	279	65
Lack of clinical guidelines for the use of cannabis for medical purposes	271	64
Risks and benefits are not sufficiently clear for potential therapeutic uses	237	56
Lack of personal knowledge/education or information regarding the use of cannabis for medical purposes	214	50
Insufficient information regarding the appropriate use of cannabis for medical purposes	212	50
Instruction from medical associations, licensing bodies, Royal College, College of Family Physicians or Canadian Medical Protective Association	201	47
Potential liability concerns	194	46
Concern about possible side effects	190	45
Uncertainty about possible interactions with other medications	167	39
Belief that cannabis is not an appropriate treatment in a specific case	141	33
Requirement to sign a declaration indicating awareness that cannabis is not an approved therapeutic under the Food and Drug Regulations	138	32
Uncertainty over whether cannabis has any medicinal value	117	27
Availability of prescription cannabinoids (e.g. nabiximols, dronabinol or nabilone)	98	23
Other	66	15

^1^Subjects may choose more than one response.

### Attitudes

When asked which health care professionals should be authorized to approve CTP, 85% reported that specialist physicians and 74% reported that family physicians should have this authority. Respondents were divided regarding nurse practitioners; 25% of respondents believed that they should and 60% believed they should not be authorized to approve CTP. The majority of respondents believed that other health care professionals should not be authorized to approve CTP (Table 4).

**Table 4:** **Beliefs about which health care professionals should have authority to approve/prescribe CTP**

Health care professional	Yes		No	
	N	% ^ 1 ^	N	%
Specialist physicians	363	85	42	10
Primary care physicians/family physicians	316	74	94	22
Nurse practitioners	108	25	256	60
Pharmacists	67	16	285	67
Naturopathic doctors	60	14	293	69
Traditional Chinese medicine practitioners	49	12	303	71
Nurses	28	7	321	75

^1^Percentages may not add up to 100% as missing data or non-responses are not included.

Seventy-one percent of respondents reported that they would feel more comfortable discussing CTP with patients/patient family members if they had more education about it, and 70% felt that with more education they would be better able to treat patients using cannabis. Comfort level with CTP was also influenced by liability protection (62%) or the availability of specific training for physicians to participate in the program (61%) (Table 5).

**Table 5:** **Factors influencing comfort level of the clinical use of cannabis for therapeutic purposes**

Factor	Agree*		Neutral		Disagree*	
	N	%	N	%	N	%
I would feel more comfortable discussing the medical use of cannabis with patients/patient family members if I had more education about it	304	71	56	13	54	13
I feel that with more education I would be better able to treat patients using medical cannabis	300	70	57	13	56	13
I would feel more comfortable authorizing medical cannabis if Health Canada offered me protection from liability	265	62	87	20	60	14
I would feel more comfortable if physicians who participated in access to CTP were required to undergo a specific training or licensing program	259	61	81	19	74	17

Percentages may not add up to 100% as missing data or non-responses are not included.

*Likert scale responses were collapsed to dichotomous outcomes: agree (strongly agree and agree) and disagree (strongly disagree and disagree).

### Educational approaches

The preferred formats for receiving educational information were peer-reviewed literature reviews on specific topics (55%), online learning programs as part of continuing medical education (54%), online resources (46%), workshops/small-group learning sessions (45%) and symposia/conferences (44%) (Table 6).

**Table 6:** **Preferred formats of educational information**

Format	n ^ 1 ^	%
Peer-reviewed literature reviews on specific topics	236	55
On-line learning programs as part of continuing medical education	230	54
On-line resources	195	46
Workshops/small-group learning sessions	192	45
Symposia/conferences	188	44
A monograph on cannabis (similar to a drug product monograph	169	40
Expert speaker tour	149	35
Grand rounds	141	33
Topic-specific reports	97	23
Mentorship/preceptorship program	79	19
Newsletter	63	15
Other	39	9

^1^Subjects may choose more than one response.

## Discussion

There is a clear need for education for health care professionals on the use of CTP. We report the results of a national survey of Canadian physicians’ perceived knowledge gaps and perceived needs concerning CTP. We found that the largest gaps between current and desired knowledge concerned dosing, the development of treatment plans, and comparisons between cannabis and existing prescription cannabinoids. There was an expressed need for better knowledge of the risks and benefits of CTP. Respondents thought that both specialists and family physicians were capable of authorising CTP; however, overall it was felt that pharmacists or naturopathic doctors should not have this authority. Concerns regarding the recreational use of cannabis masquerading as medical use was common among respondents. Respondents reported that their comfort level in including CTP in their practice would increase with additional education, and reported their educational needs would be best met with focused literature reviews, online, and small group continuing medical education activities.

Ranking perceived knowledge levels on several related topics allows a comparison of ‘what is’ and ‘what should be’ regarding perceived educational needs, and enables the quantification of perceived knowledge gaps. Describing and ranking the perceived knowledge gaps for several CTP subtopics enables a comparison of gaps between topics, and enables the identification of strategies to reduce the gaps [13,14]. Based on our data, Canadian physicians perceive their current knowledge level on CTP to be low, while desiring a high knowledge level, consistent with other reports that physicians desire education on CTP [9,10]. More specifically, we identified the lowest perceived knowledge levels, and largest knowledge gaps, to be regarding hands-on clinically relevant CTP subtopics, including; dosing and treatment plans, comparing between cannabinoids, and Canadian CTP regulations. In contrast, we identified higher perceived current knowledge levels, and smaller knowledge gaps, to be regarding more theory-based CTP subtopics, including; the mechanism of action of cannabis, potential risks, and potential benefits. This suggests that practical hands-on style information should be prioritized.

Two interesting discrepancies emerge from responses about experiences with the clinical use of CTP. Firstly, patients initiate most of the discussions about CTP, and physicians feel they have insufficient information and lack guidance on the topic. Few guidelines exist for CTP [15,16], while Canadian physicians have reported that clinical practice guidelines would be useful or very useful [3]. Secondly, only one third of respondents had signed a patient’s application to possess CTP through the federally regulated program, yet two thirds reported having patients using CTP. It is possible that respondents had patients who had received CTP from another physician. The discrepancy between the prevalence of self reported use of CTP (48%) and the proportion of patients with legal access through Canada’s federal program (32%) has been reported previously in certain populations [17,18].

The highest reported obstacle to CTP was found to be a concern that patients are actually seeking cannabis for recreational purposes, supporting similar results from previous reports [4,9,10]. Patients report experiencing a lack of trust by health care providers, and suggest that it is due to the stigma associated with cannabis as an illegal recreational drug [17]. Such views may be a result of the blurred lines between therapeutic and recreational use reported in the media [19]. Another potential cause for this distrust of patient motivation for CTP use stems from the fact that some of the patient populations in which cannabis may be a potential therapeutic option, such as chronic pain, HIV/AIDS, and mental health issues, are already stigmatized [17]. Canadian patients using CTP have reported the stigma associated with cannabis negatively impacts their relationship with health care providers, creating a barrier to receiving the health care they need, and increased levels of physician education may be a means to decrease the stigma [17]. It may be the case that negative attitudes and values about cannabis in general influence potential therapeutic uses, as has been reported in other controversial therapeutic areas like methadone maintenance therapy [20]. Finally, the demographic overlap (young white males) between medical cannabis users and recreational users also adds to the potential for physician mistrust of the real medical ‘necessity’ for CTP [21,22].

Our survey has several potential limitations. The low number of responses creates a potential selection bias, which affects our ability to generalize findings, and which may not be representative of Canadian physicians overall. A true response rate is impossible to calculate, as the denominator is unknown; we do not know how many different physicians received or saw the survey. The low number of overall responses may be due, in part, to the lack of monetary compensation for participation [23], a large number of similar requests, lack of time, or hesitancy to enter answers into a survey on this topic. Another contributing factor to the low number of responses may be the source of the invitations; other non-medical-college distributed surveys have reported similarly low response rates from Canadian physicians [24], whereas surveys sent by provincial or national medical colleges or associations have reported response rates around 20% or higher [9,10,20,25]. An additional limitation is that a number of recipients of email invitations were based on lists of previous participants in CTP-related continuing medical education programs, or those who had expressed interest in topic of CTP to a variety of sources. This may have selected participants with specific interest in CTP or who had already had some education on this topic. However the number of responses received is similar to other recent physician surveys on CTP including the 607 responses received by the Canadian Medical Association (CMA) [10], and 520 responses to the Colorado physician survey [9]. Finally, this study is limited by the focus on perceived needs and did not include an assessment of unperceived needs; we did not determine whether high levels of perceived knowledge were in fact truly high levels of knowledge based on external evaluation. Triangulating perceived needs with unperceived needs should be the focus of further research.

Our results support the need for further medical education and training on CTP [9]. In alignment with expressed needs for more information on CTP, physicians reported that focused peer-reviewed summaries on specific CTP sub-topics would be helpful in enhancing their knowledge on this area. The preference for online education is consistent with trends towards e-learning in the health professions [26]. Additionally, studies have reported that educational interventions that enhance competencies and skills have a direct influence on improvement of patient outcomes [27].

## Conclusions

Cannabis is not a pharmaceutical product and thus has not taken a traditional route into the physicians’ toolbox of potential therapies and has not undergone the same rigorous testing demanded by Health Canada of pharmaceutical medications. However, research on cannabinoids and the endocannabinoid system has increased over the past 20 years. This growing body of research needs to be translated into resources to address physicians’ professional knowledge and practice gaps to enable them to make more informed decisions about CTP. The new MMPR allows nurse practitioners and physicians to authorize patients’ legal access to CTP. The knowledge gaps and educational needs among nurse practitioners is yet to be described. Future needs assessments among physicians and nurse practitioners should evaluate unperceived needs in addition to perceived needs. The transition to the new federal regulations provides an opportunity to develop and implement evidence-based education for physicians and nurse practitioners that should address the existing perceived knowledge gaps we describe, and to evaluate the effectiveness of such strategies on clinical practice and, ultimately, on health outcomes.
